# Are ipsilateral breast tumour invasive recurrences in young (⩽40 years) women more aggressive than their primary tumours?

**DOI:** 10.1038/sj.bjc.6603991

**Published:** 2007-09-18

**Authors:** B Sigal-Zafrani, M A Bollet, G Antoni, A Savignoni, A Vincent-Salomon, J-Y Pierga, R Salmon, X Sastre-Garau, A Fourquet

**Affiliations:** 1 Department of Pathology, Institut Curie, Paris, France; 2 Department of Radiation Oncology, Institut Curie, Paris, France; 3 Department of Biostatistics, Institut Curie, Paris, France; 4 Department of Medical Oncology, Institut Curie, Université René Descartes Paris 5, Paris, France; 5 Department of Surgery, Institut Curie, Paris, France

**Keywords:** breast cancer, local recurrence, breast-conserving treatment, young age

## Abstract

The characteristics of ipsilateral breast tumour recurrences (IBTRs) relative to those of their primary tumours (PTs) remain scarcely studied. Of 70 young (⩽40 years) premenopausal women with IBTRs, we studied a series of 63 with paired histological data. Median follow-up since IBTR was 10 years. Rates of histological types, grades or hormonal receptors were not significantly different in PTs and in IBTRs. The concordance between IBTRs and their PTs was good for histological types. IBTRs with conserved histological types tended to occur more locally, but not significantly sooner than others. These IBTRs had good concordance for hormone receptors. In discordant cases there were as many losses as appearances of the receptors. The concordance was weak for grades, with equivalent numbers of IBTRs graded lower as higher than their PTs. The 10-year overall survival rate was 70%. Neither the conservation of histological type, location, nor of the two combined were associated with deaths. Early (<2 years) IBTRs, tended to be associated with poorer survival (HR=2.24 (0.92–5.41); *P*=0.08). IBTRs did not display features of higher aggressiveness than PTs. Neither clinical nor histological definition of a true recurrence could be established other than the conservation of the histological type.

Breast-conserving treatments of early stage breast cancer exposes the patient to the risk of ipsilateral breast tumour recurrence (IBTR). The most important prognostic factor for local recurrence is the young age of the patient (Vrieling *et al*, 2003) and this remains true among young (<40 years old), premenopausal women treated either by surgery first (Bollet *et al*, 2007) or by neoadjuvant chemotherapy (Oh *et al*, 2006). Many questions remain unanswered concerning the real nature of these ipsilateral breast tumour recurrences. We shall examine how different they are from primary tumours and whether there are clinical or histological factors to help distinguish between a re-growth of malignant cells not removed by surgery and not killed by radiotherapy (also called a true recurrence, TR) and a *de novo* malignancy arising from mammary epithelial cells of residual breast tissues (also known as new primary tumours, NP) (Haffty *et al*, 1993). Some have implied that ipsilateral breast tumour recurrences should display features of, at least as much aggressiveness (differentiation (Huang *et al*, 2002), ploidy (Haffty *et al*, 1993) and percentage of invasiveness (Haffty *et al*, 1993; Smith *et al*, 2000; Huang *et al*, 2002)) to qualify as true recurrences. We shall see whether biological evidence can be found to support this definition. Finally, we shall investigate whether the characteristics of ipsilateral breast tumour recurrences are associated with prognosis.

## PATIENTS AND METHODS

Out of a previously described series of 209 premenopausal women, younger than 40 years old, treated at the Institut Curie between 1985 and 1995 for early breast cancers (clinical T1-2, N0-1; Sobin and Wittekind, 2002) with primary breast-conserving surgery and followed over a long period (median follow-up of 12 years), 70 patients were diagnosed with local recurrences (Bollet *et al*, 2007). We consider here a group of 63 patients for whom histological and immuno-histochemical data were available for both the primary tumour and the local relapse (some patients were not treated for their local relapse at the Institut Curie). We report here a study about the similarities between primary breast cancers and their local relapses in terms of histological and immuno-histochemical features according to the site of relapse and the time-lapse before relapse. Median age at diagnosis was 36 years (23–40) with 57% of patients (36 patients) 35 year old or younger and 43% between 36 and 39 years old (27 patients). All patients were premenopausal at the time of the initial treatment. A family history indicating that at least one first- or second-degree relative with a history of breast cancer was present in 71% of patients (45 patients), absent in 27% (17 patients) and unknown in 2% (1 patient).

Patients' characteristics were as follows: clinical tumour stage (Sobin and Wittekind, 2002) was T1 for 70% (44 patients) and T2 for 30% (19 patients). N stage was N0 for 73% (50 patients), N1 for 21 % (13 patients).

All the specimens were reviewed by the same qualified breast pathologist (BSZ). Histological classification of the infiltrating carcinomas was done according to the World Health Organization criteria and histological grades were scored according to Elston and Ellis (1991). The mitotic index was determined according to the number of mitoses per 10 high power fields (each field corresponded to a surface of 3.3 mm^2^), as low when <11, moderate when (11–22) and high when >22 (Vincent-Salomon *et al*, 2004). Hormonal receptors were positive if they showed staining of either oestradiol receptors (ER) or progesterone receptors in at least 10% of invasive tumour cells by immuno-histochemistry.

Treatments of the primary tumour consisted, for all patients, in surgery with breast-conserving procedures as the first treatment. The quality of the surgical margin was stated as wide (>3 mm) in 40 patients (63%), close (⩽3 mm) in 14 (22%), involved with ductal carcinoma *in situ* in four (6%), involved with invasive carcinoma in five (8%). The reasons for the absence of re-excision were not always specified. When they were, it was because of the patient's choice not to undergo a new surgical procedure that could have been a mastectomy.

All patients received post-operative radiotherapy with a median dose of 54 Gy (45–62) to the breast. A boost to the tumorectomy bed was performed for 68% (36 patients) of the women with a median dose of 16 Gy (2–25). The median total dose to the tumorectomy bed was 62 Gy (52–76). There was no protocol to boost all young patients with negative surgical margins at that time and some of the patients reported in this series were accrued in the EORTC boost trial that randomised from 1989 to 1996 between boost and no boost (Bartelink *et al*, 2001; Vrieling *et al*, 2003; Antonini *et al*, 2006). In the case of positive surgical margins, a radiotherapy boost of generally 20–28 Gy was added to the whole-breast irradiation. For patients who were not participating in the European Organisation for Research and Treatment of Cancer randomised trial, a boost of 10–16 Gy was added in the case of aggressive histological features (unsatisfactory margins, high histological grade, high proliferation index, absence of hormone receptors).

Systemic treatments were given in 30% (63 patients) of the women. At the time of treatment, the protocol for premenopausal women consisted of anthracycline-based polychemotherapy (usually six cycles of 5-fluorouracil, doxorubicin, cyclophosphamide) without hormone therapy.

### Statistics

Comparisons of pathological characteristics between primary tumours and ipsilateral breast tumour recurrences were performed by McNemar χ^2^ paired analysis that tests the hypothesis that, among discordant cases, the evolution was more often of one kind than another. For instance, when applied to oestradiol receptors, there was a significant difference between the numbers of cases with a loss of ER and those with an appearance of ER (ER− in PT and ER+ in IBTR). When the analysed pathological feature had more than two classes, analysis was downsized to a 2 × 2 table.

Differences in qualitative assessments between the two groups of patients were tested by Fisher's exact test or the χ^2^-test, while differences in quantitative measurements were tested by either the Student's *t*-test or Kruskal–Wallis test (depending on the distribution characteristics). Median follow-up was calculated using the reverse Kaplan–Meier estimator (Kaplan and Meier, 1958). Overall survival was defined as the lapse of time from the date of ipsilateral breast tumour recurrence to the date of death. Distant relapse-free survival was defined from the date of ipsilateral breast tumour recurrence to the date of diagnosis of distant relapse or death. Overall and distant relapse-free survivals were estimated with the Kaplan–Meier technique (Kaplan and Meier, 1958). Cox proportional hazards regression models were used to estimate the effects of the type of ipsilateral breast tumour recurrences on prognosis. Estimates of effects are presented as hazard ratios with their associated 95% confidence intervals. A higher hazard ratio is associated with an increased risk of event (death or metastasis).

All concordance tests were calculated according to Cohen's Kappa. A kappa value <0.5 is considered weak, 0.5–0.7 fair, and >0.7 is considered very good concordance. The level of statistical significance was 0.05. The concordance tests were always assessed for the whole group of patients and in the two different subpopulations according to the lapse of time of occurrence of the local relapse from the first treatment of the primary tumour (within the first 5 years or later) or to the site of the local recurrence with respect to the primary tumour (same quadrant or different quadrant).

## RESULTS

Median time-lapse between the primary tumour and its ipsilateral breast tumour recurrence was 43 months (3–158). Median follow-up since ipsilateral breast tumour recurrence was 10 years (1–18).

Histological details of both the primary tumour and its ipsilateral breast tumour recurrence are given in Table 1. In summary, the rates of histological types, grades (and their components) or hormonal receptors were not statistically different in the primary tumours from the ipsilateral breast tumour recurrences.

Ipsilateral breast tumour recurrences occurred in the index quadrant, the one where the primary tumour had arisen, in 70% (44 patients), in a different quadrant in 27% (17 patients) and in 3% of cases (two patients) the relative locations were unknown. The mean time-lapse before occurrence of the ipsilateral breast tumour recurrence was not significantly different in the case of a recurrence at the index quadrant or not (respectively 55 *vs* 53 months; *P*=0.8). The rate of ipsilateral breast tumour recurrences occurring within the first 2 years was not significantly higher for those that had recurred at the index quadrant than for the others (18 *vs* 12%; *P*=0.72).

### Concordance tests

#### Histological types of infiltrating carcinomas

As represented in Table 2 there was a good concordance with 94% of cases (59 patients out of 63) with conservation of the histological types κ 0.72. The ipsilateral breast tumours that shared their primary tumour's histological types tended to occur more locally than those with a different histological type (rate of recurrence in the index quadrant of 75 *vs* 25%; *P*=0.07) but not statistically sooner (median time-lapse before occurrence of 41 *vs* 71 months; *P*=0.36).

In 45 cases (71%) ipsilateral breast tumour recurrences shared both the same histological type and the same location (index quadrant) with their primary tumours. These ipsilateral breast tumour recurrences did not occur significantly sooner than the others (median time lapse of 38 *vs* 54 months; *P*=0.34).

In 33 cases out of 61 cases for which data were available (52%), the ipsilateral breast tumour recurrences had occurred not only with a conserved histological type, but also with features of equal or lesser differentiation (loss of hazard ratio, higher histological grade) than their primary tumours. These cases did not occur significantly neither more locally (rate of recurrence in the index quadrant of 81 *vs* 61%; *P*=0.15) nor sooner (median time lapse of 37 *vs* 51 months; *P*=0.19) than others.

#### Sub-analysis of IBTR with conserved histological types (59 pairs)

As represented in Table 3, provided that only conserved histological types were studied, the concordance between primary tumours and their local recurrences was weak for progesterone (κ 0.47), fair for oestradiol (κ 0.52) and good for hormone (κ 0.85) receptors. In the case of discordant cases, there was no statistically significant difference between changes, whether loss or appearance of the receptors.

Of the 54 pairs for which both the primary tumours and the ipsilateral breast tumour recurrences were histologically graded, the grades remained the same in 21 patients (39%), weak concordance (κ 0.03). There was no statistically significant difference between patients for whom the IBTR was graded higher and those for whom it was graded lower than the primary tumour (17 *vs* 16 patients; *P*=0.86).

### Prognostic implications for the conservation of location, histological type or both on overall survival

Rates of 5- and 10-year overall survivals were respectively 79% (69–90) and 70% (59–84).

Neither the conservation of the histological type in the ipsilateral breast tumour recurrence (hazard ratio=1.27 (0.29–5.56); *P*=0.75), nor the conservation of location with ipsilateral breast tumour recurrence occurring at the index quadrant (hazard ratio=1.13 (0.43–2.95); *P*=0.8), nor the two combined, that is conservation of both histological type and location versus the others (hazard ratio=1.07 (0.43–2.7); *P*=0.88) nor those with conserved histological types and equal or lesser differentiation (hazard ratio=2.08 (0.85–5.2); *P*=0.11) were statistically associated with deaths. Early ipsilateral breast tumour recurrences, defined as occurring within the first 2 years, tended to be associated with a higher rate of death (hazard ratio=2.24 (0.92–5.41); *P*=0.08) than late recurrences. When comparing both the tumours and their ipsilateral breast tumour recurrences according to the free interval with a cutoff at 2 years, the only statistically significant differences were that primary tumours associated with early ipsilateral breast tumour recurrences (IBTRs) were more proliferating than the others and that early IBTRs (occurring within the first 2 years) had a higher degree of nuclear polymorphism than late IBTRs, as shown in Table 4.

## DISCUSSION

The occurrence of an ipsilateral breast tumour recurrence is associated with poor prognosis because of the potential risk of lethality from its propensity to yield metastases (Clarke *et al*, 2005).

In this study of a homogeneous group of young (⩽40 years old) patients, we did not find that, *per se,* ipsilateral breast tumour recurrences display features of higher aggressiveness than primary tumours (Table 1). All features were highly comparable between the two groups of tumours.

In some cases, the occurrence of an ipsilateral breast tumour recurrence can also reflect the evolution of the primary tumour and thus its resistance to the adjuvant therapies used in the first instance: that is radiotherapy with or without systemic treatments. These ipsilateral breast tumour recurrences are also called true recurrences, in opposition to new primaries. Their definition according to clinical and histological criteria remains a wild dream. All authors agree on a basic definition of a new primary that is, an ipsilateral breast tumour recurrence with a different histological type from its primary tumour (Haffty *et al*, 1993; Touboul *et al*, 1999; Smith *et al*, 2000; Huang *et al*, 2002; Komoike *et al*, 2005). Our series showed a good concordance of the histological type in the ipsilateral breast tumour recurrence.

Most authors have also proposed that location would help distinguish the two - with ipsilateral breast tumour recurrences occurring away from the index quadrant more prone to be new primaries (Fisher *et al*, 1986; Haffty *et al*, 1993; Smith *et al*, 2000; Huang *et al*, 2002). This was confirmed in our series where ipsilateral breast tumour recurrences that had conserved their primary tumours' histological types tended to arise more often in the index quadrant than others. When addressing only ipsilateral breast tumour recurrences with conserved histological types, we showed a good concordance with their primary tumours in terms of hormonal receptors but the absence of any concordance in terms of histological grade and its components. Bearing in mind the inter-observer disagreement (Frierson *et al*, 1995; Robbins *et al*, 1995), there is always the concern that it could reflect an intra-observer variability in the definition of the histological grade. The question remains whether the assumption that the definition of true recurrences could rely on the possibility that true recurrences show features of lesser differentiation than their primary tumours. To address it, we first looked at discordant cases in terms of histological grade. There was no sign that the evolution was more prone to be towards more aggressiveness than less. Secondly, ipsilateral breast tumour recurrences with conserved histological types with features of equal or lesser differentiation than their primary tumours did not occur significantly more locally.

The arguments to oppose this theoretical assumption (that true recurrences display at most equal but not higher differentiation than their primaries) are twofold. First, some adjuvant treatments such as chemotherapy could electively kill the most undifferentiated clones from the primary tumour and thus select those of higher differentiation, resistant to chemotherapy. The equivalent has already been shown in some studies where either the clonal heterogeneity found in the primary tumours (Teixeira *et al*, 1996) or the amount of their chromosomal alterations (Kuukasjarvi *et al*, 1997) were reduced in their metastases. Second, the chronological order of the discovery of two breast cancers with clonal homology does not forcefully reflect the paternity of the first over the second because a tumour with few alterations can be occult for years following the removal of a more deranged derivative. This has been inferred in the case of bladder cancer from the study of several tumours removed from the same patient over a long period of time (van Tilborg *et al*, 2000). Beside these arguments, the absence of a trend for a progression of grade between different stages of the breast cancer has already been shown by Millis *et al* (1998) both from ductal carcinoma *in-situ* to invasive ductal carcinoma and from invasive ductal carcinoma to axillary lymph nodes, local (ipsilateral breast tumour recurrences excluded) or distant recurrences.

Contrary to others (Haffty *et al*, 1993; Goldstein *et al*, 2005; Vicini *et al*, 2007), we found no indication that tumours more prone to be true recurrences because of conserved histological type, location or both, recur sooner or are deadlier than the others. The same applied to the conservation of histological type and features of equal or lesser differentiation. On the other hand, we confirmed the notion that early recurrences, that is occurring within the first 2 years, are associated with more deaths than late ones (Fourquet *et al*, 1989; Fisher *et al*, 1991; Whelan *et al*, 1994; Veronesi *et al*, 1995; Haffty *et al*, 1996; Fredriksson *et al*, 2002). In our study the group of patients with early recurrences had primary tumours significantly more proliferating and ipsilateral breast tumour recurrences with a higher degree of nuclear polymorphism than the group of patients with late recurrences.

The main drawback of this study is the relatively small size of our series under-powering the possibility of revealing real associations between the assessed factors. We conclude however that the definition of true recurrences according to clinical and histological criteria should rely solely on the conservation of the histological type with the added information brought by the location. There is no indication that true recurrences should have features of equal or lesser differentiation than their primary tumours.

However, because of the overwhelmingly high rate of the ductal histological type among breast cancers, there is need to improve the definition of true recurrences by using new biological tools of clonal relation such as pan-genomic profiles (Waldman *et al*, 2000), loss of heterozygosity (Schlechter *et al*, 2004; Vicini *et al*, 2007), p53 mutation analysis (van der Sijp *et al*, 2002) or the inactivation of the X chromosome (Shibata *et al*, 1996). Caution is however still needed as some of these techniques conclude that only 65% of clonally related ipsilateral breast tumour recurrences have the same histological types as their primary tumours (Vicini *et al*, 2007).

We hope that a better distinction of true recurrences will open new perspectives. So far, very little is known about the differential or similarity of the pan-genomic expression or nature of both new primaries and IBTRs. Kreike *et al* (2006) reported a gene expression analysis of 18 000 cDNAs on nine pairs of primary breast cancer with their ipsilateral breast recurrences in women who were younger than 51 years old at the time of their initial breast-conserving therapy. Paired-data analysis showed no set of genes that was consistently different in expression between primary tumours and local recurrences. Better focusing on true recurrences only and comparing them in detail with their primary tumours, could hopefully reveal possible clonal selections of cells endowed with attributes of either radio-resistance or aggressiveness. Another route that has still scarcely been explored is the search for a transcriptomic signature to predict the risk of local recurrence after breast-conserving treatment (Kreike *et al*, 2006; Nuyten *et al*, 2006). Here also, a better distinction between new primaries and true recurrences is needed to perform a supervised study according to the occurrence of true recurrences only and not of all ipsilateral breast tumour recurrences.

## CONCLUSION

Ipsilateral breast tumour recurrences did not display features of higher aggressiveness than primary tumours. There was no indication that tumours more prone to be true recurrences because of conserved histological type, location or both, recur sooner or are deadlier than the others. No clinical nor histological definition of a true recurrence could be established other than the conservation of the histological type.

## Figures and Tables

**Table 1:** Patients' and tumours' characteristics

	**Primary tumour**		**IBTR**		
	***N***	**%**	***N***	**%**	***P***
*Infiltrating carcinoma £*	DM=0		DM=0		1
Ductal	55	87	55	87	
Lobular	4	6	6	10	
Other	4	6	2	3	
					
*Histological grade*	DM=4		DM=3		1
1	22	37	16	27	
2	23	39	35	58	
3	14	24	9	15	
					
*Tubule formation*	DM=0				1
1.2	15	24	16	25	
3	48	76	47	75	
					
*Nuclear polymorphism*	DM=0		DM=0		1
1	10	16	9	14	
2	42	67	43	68	
3	11	17	11	17	
					
*Mitoses per 10 HPF*	DM=4		DM=3		0.48
[0-10]	40	68	40	67	
[11-21]	11	19	14	23	
⩾22	8	14	6	10	
					
*Oestradiol receptors*	DM=0		DM=0		0.43
Positive (⩾10 %)	41	65	45	71	
Negative	22	35	18	29	
					
*Progesterone receptors*	DM=0		DM=0		1
Positive (⩾10%)	42	67	41	65	
Negative	21	33	22	35	
					
*Hormone receptors*					1
Positive (either ER or PR ⩾10 %)	49	78	50	79	
Negative (both ER and PR <10%)	14	22	13	21	

ER=oestradiol receptors; HPF=high power field; IBTR=ipsilateral breast tumour recurrences; PR=progesterone receptor.

**Table 2:** Comparison between pathological characteristics between PT and their ipsilateral breast tumour recurrences

	**Ipsilateral breast tumour recurrences**		
**Histological type of the infiltrating carcinomas paired *χ*^2^ (McNemar test)=1 *κ*=0.72**	**Ductal**	**Lobular**	**Mucinous**
*Primary*			
Ductal	53	2	0
Lobular	0	4	0
			
*Tumours*			
Mucinous	0	0	2
Other	2	0	0

PT=primary tumours.

**Table 3:** Comparison of pathological characteristics between PT and their ipsilateral breast tumour recurrences provided that they share the same histological types (59 pairs of PT/IBTR)

	**Ipsilateral breast tumour recurrence**		
**Oestradiol receptors McNemar's test=0.39 κ=0.52**	**Negative**	**Positive**	
*Primary tumour*			
Negative	12	8	
Positive	4	35	
			
	**Ipsilateral breast tumour recurrence**		
**Progesterone receptors McNemar's test=1 κ=0.47**	**Negative**	**Positive**	
*Primary tumour*			
Negative	13	7	
Positive	7	32	
			
	**Ipsilateral breast tumour recurrence**		
**Hormone receptors McNemar's test=1 κ=0.85**	**Negative**	**Positive**	
*Primary tumour*			
Negative	11	2	
Positive	1	45	
			
	**Ipsilateral breast tumour recurrence**		
**Histological grade (DM=5) McNemar's test=0.86 κ=0.03**	**Grade 1**	**Grade 2**	**Grade 3**
*Primary tumour*			
Grade 1	7	12	1
Grade 2	7	11	4
Grade 3	0	9	3
			
	**Ipsilateral breast tumour recurrence**		
**Mitotic index (DM=5) McNemar's test=0.63 κ=0.33**	**Low**	**Moderate**	**High**
*Primary tumour*			
Low	31	3	3
Moderate	4	5	1
High	3	3	1
			
	**Ipsilateral breast tumour recurrence**		
**Nuclear polymorphism (DM=0) McNemar's test=0.61 κ=0.33**	**Low**	**Moderate**	**High**
*Primary tumour*			
Low	5	3	1
Moderate	2	34	5
High	0	4	5
			
	**Ipsilateral breast tumour recurrence**		
**Tubule formation (DM=0) McNemar's test=1 κ=0.12**	**Low**	**Moderate**	**High**
*Primary tumour*			
Low	0	2	1
Moderate	0	4	8
High	1	9	34

**Table 4:** Comparison of pathological features of primary tumours and of IBTR according to the free interval between the two (within or after 2 years)

	**Primary tumour**					**IBTR**				
	**Early (*N*=52)**		**Late (*N*=11)**			**Early (*N*=52)**		**Late (*N*=11)**		
	***N***	**%**	***N***	**%**	***P***	***N***	**%**	***N***	**%**	***P***
*Infiltrating carcinoma*					0.15					0.6
Ductal	47	90	8	73		46	88	9	82	
Lobular	2	4	2	18		4	8	2	18	
Other	3	6	1	9		2	4	0	0	
										
*Histological grade*					0.18					0.2
1	20	41	2	20		15	30	1	10	
2	20	41	3	30		29	58	6	60	
3	9	18	5	50		6	12	3	30	
										
*Tubule formation*					0.06					0.8
1.2	15	29	0	0		14	27	2	18	
3	37	71	11	100		38	73	9	82	
										
*Nuclear polymorphism*					0.13					**0.005**
1	8	15	2	18		8	15	1	9	
2	37	71	5	45		39	75	4	36	
3	7	13	4	36		5	10	6	55	
										
*Mitoses per 10 HPF* a					**0.02**					0.15
[0–10]	37	76	3	30		36	72	4	40	
[11–21]	7	14	4	40		10	20	4	40	
⩾22	5	10	3	30		4	8	2	20	
										
*Oestradiol receptors*					0.5					0.3
Positive	35	67	6	55		39	75	6	55	
Negative	17	33	5	45		13	25	5	45	
										
*Progesterone receptors*					0.8					0.18
Positive	34	65	8	73		36	69	5	45	
Negative	18	35	3	27		16	31	6	55	
										
*Hormone receptors*					0.7					0.22
Positive	41	79	8	73		43	83	7	64	
Negative	11	21	3	27		9	17	4	36	

IBTR=ipsilateral breast tumour recurrences.

a HPF=high power field.

Bold values are statistically significant *P*-values.
